# Exercise Dependence Psychopathology Is Associated with Differential Biochemical Correlates at Admission in Anorexia Nervosa: An Exploratory Cross-Sectional Study

**DOI:** 10.3390/brainsci16101114

**Published:** 2026-10-15

**Authors:** Francesco Monaco, Annarita Vignapiano, Ernesta Panarello, Stefania Landi, Felice Spista, Rossella Bonifacio, Daniela Falchetta, Alessandra Marenna, Maria Laura Mignone, Luca Steardo, Gennaro Sosto, Giulio Corrivetti

**Affiliations:** 1 Department of Mental Health, ASL Salerno, 84121 Salerno, Italy; fmonaco1980@gmail.com (F.M.);; 2 EBRIS— European Biomedical Research Institute of Salerno, 84131 Salerno, Italy; 3 Department of Psychology, University of Campania “Luigi Vanvitelli”, 80100 Caserta, Italy; 4 General Management, ASL Salerno, 84121 Salerno, Italy

**Keywords:** anorexia nervosa, exercise dependence, EDS-R, FT3, LDH, ALT, low T3 syndrome, biochemical parameters, residential treatment, compulsive exercise

## Abstract

**Highlights:**

**What are the main findings?**

- In 164 patients with anorexia nervosa (first admissions only; 13 repeat-admission rows excluded from 177 total) admitted to a specialist residential unit, higher EDS-R scores showed adjusted cross-sectional associations with lower FT3 levels (β = −0.040, *p* = 0.016) and higher LDH levels (β = +14.56, *p* = 0.009), independent of BMI, age, AN subtype, and illness duration. The EDS-R association with low-T3 syndrome was confirmed in the logistic model (OR = 1.290, 95% CI [1.043–1.596], *p* = 0.019).
- AN subtype was not an independent predictor in the adjusted models; a post hoc EDS-R-by-subtype interaction analysis in the logistic model yielded *p* = 0.005 (interaction OR = 0.474, 95% CI [0.282, 0.797], N = 144) for the EDS-R × AN subtype term, indicating that the EDS-R association with low-T3 syndrome is significantly stronger in AN-R than AN-BP.

**What are the implications of the main findings?**

- These exploratory findings suggest that exercise dependence psychopathology may be associated with differential biochemical patterns at residential admission in anorexia nervosa, warranting prospective replication before any clinical interpretation.
- Prospective studies are needed before these findings can inform clinical practice.

**Abstract:**

Background: We investigated, in an exploratory cross-sectional design, whether EDS-R score at admission showed adjusted cross-sectional associations with FT3, LDH, and ALT in patients with anorexia nervosa (AN) in residential treatment. Methods: A total of 164 patients with AN (first admission per patient) (AN-R: n = 122; AN-BP: n = 42; mean BMI 15.7 ± 1.7 kg/m^2^; 98.2% female) admitted consecutively to a specialist residential unit were included. Biochemical parameters were assessed within 48 h of admission using fully automated, internationally standardised analytical methods. FT3 was measured by electrochemiluminescence immunoassay (ECLIA) on automated immunoassay platforms, in accordance with standard immunometric procedures. ALT and LDH were measured by kinetic enzymatic (UV) methods following IFCC recommendations, using high-throughput clinical chemistry analysers. All analyses were performed in the core laboratory (Corelab) of the ASL Salerno hospital network, with internal and external quality control programmes in place. Exercise dependence severity was measured using the EDS-R Multivariable OLS and logistic regression analyses were conducted, adjusting for age, BMI, disease duration, and AN subtype. Results: Among 16 biochemical parameters examined, FT3 and LDH showed the largest Spearman correlations with EDS-R score, though neither survived Bonferroni correction for 16 simultaneous tests (threshold: *p* < 0.0031) (FT3: ρ = −0.224, *p* = 0.007; LDH: ρ = +0.244, *p* = 0.006). In multivariable regression, EDS-R score was independently associated with lower FT3 (β = −0.040, 95% CI [−0.072, −0.008], *p* = 0.016, n = 144) and higher LDH (β = +14.56, 95% CI [3.78, 25.35], *p* = 0.009, n = 124), while BMI was not significant in either model. By contrast, BMI was the sole significant predictor of ALT (β = −0.078, 95% CI [−0.136, −0.021], *p* = 0.008, n = 154), with the EDS-R score being non-significant (β = 0.019, *p* = 0.604). Binary logistic regression confirmed an association between EDS-R score and low-T3 syndrome (FT3 < 2.30 pg/mL): OR = 1.290 (95% CI [1.043–1.596], *p* = 0.019). Conclusions: In this exploratory cross-sectional study, EDS-R score showed adjusted associations with lower FT3 and higher LDH, but not ALT, at residential admission in AN, independent of BMI, age, AN subtype, and illness duration. These hypothesis-generating, cross-sectional findings cannot be interpreted as establishing prediction, causation, biomarker validity, or clinical utility. Prospective replication is required before any clinical implications can be considered.

## 1. Introduction

Anorexia nervosa (AN) is a severe psychiatric disorder characterised by persistent restriction of energy intake, intense fear of weight gain, and distorted body image, and carries the highest standardised mortality ratio among all psychiatric conditions [1,2]. Alongside the nutritional and psychological dimensions of the disorder, compulsive or excessive exercise, variously termed exercise dependence, compulsive exercise, or hyperactivity, represents one of the most prevalent, clinically refractory, and prognostically significant behavioural features of AN [3,4]. Estimates suggest that compulsive exercise coexists with AN in a substantial proportion of patients [5,6], functioning not merely as a weight-control strategy but as a primary mechanism of affective regulation and anxiety modulation [6,7,8]. Its presence at admission has been associated with earlier illness onset [9], more severe eating disorder psychopathology [10], longer hospitalisation [11], and higher post-discharge relapse rates [12].

When a severely underweight patient is admitted to a residential treatment unit, clinicians are confronted with a deeply altered biochemical profile whose determinants are typically attributed to nutritional deprivation alone. Foremost among these alterations is elevation of hepatic transaminases, alanine aminotransferase (ALT), predominantly ascribed to starvation-induced hepatocyte autophagy, a process in which the liver catabolises its own parenchymal cells to yield gluconeogenic substrates, releasing transaminases as a by-product [13,14,15,16]. The critical implication is that hepatic enzyme elevation in AN is commonly associated with starvation-related hepatic injury, while contributions from other factors cannot be fully excluded.

High-intensity repetitive activity in a state of severe energy deficit may involve partly different physiological pathways. High-volume repetitive activity in severe energy deficit, a well-documented phenomenon in AN [17], may be associated with cellular breakdown and release of intracellular enzymes, including LDH, into systemic circulation [18,19,20,21]. This potential distinction, hepatic enzyme elevation associated with starvation versus LDH elevation associated with compulsive movement, has rarely been examined in clinical AN samples using a validated behavioural measure of exercise dependence.

A third axis is implicated by the convergence of starvation and compulsive exercise: the hypothalamic–pituitary–thyroid axis. In response to prolonged energy deficit, peripheral conversion of thyroxine to the metabolically active free triiodothyronine (FT3) is suppressed, producing the low-T3 syndrome, an adaptive metabolic down-regulation system well documented in AN [22,23,24,25,26,27]. Whether exercise dependence psychopathology is associated with an independent acceleration of this suppression, beyond what nutritional compromise alone produces, remains unresolved and is one of the questions this study seeks to explore.

The primary aim of this study was to exploratorily assess whether EDS-R total score at admission showed adjusted cross-sectional associations with three a priori defined biochemical parameters, FT3, LDH, and ALT in patients with AN admitted to a specialist residential unit, over and above age, BMI, disease duration, and AN subtype. A secondary aim was to examine whether the pattern of these associations differed across biochemical markers, with particular attention to whether EDS-R score and BMI show contrasting associations across the three outcomes.

## 2. Methods

### 2.1. Study Design and Setting

This was a retrospective cross-sectional study conducted at the Regional Residential Centre for Eating Disorders “Mariconda”, ASL Salerno, Italy. Data were collected from consecutive admissions between January 2018 and June 2026. Thirteen repeat admissions by 12 patients were identified and excluded; only the first admission per patient was retained for analysis. The study was conducted in accordance with the Declaration of Helsinki. The analysis used retrospectively extracted, fully anonymised data collected as part of routine clinical care, with no additional research procedures. No formal ethics committee approval number or written waiver was available for this retrospective analysis; no institutional document or administrative determination from ASL Salerno formally establishing the basis for this secondary analysis without ethics committee review is available. Data-protection legislation does not constitute ethical approval or exemption. Data were handled in accordance with Italian Legislative Decree No. 196/2003 and GDPR Regulation EU 2016/679.

### 2.2. Participants

Eligible patients were adults and adolescents admitted with a primary DSM-5 diagnosis of anorexia nervosa (restricting type, AN-R, or binge-purge type, AN-BP) [1]. Patients with bulimia nervosa, binge eating disorder, ARFID, or other specified feeding and eating disorders were excluded from the primary analysis: the distinct nutritional context of these diagnoses (particularly the normal-range BMI typical of BN) would introduce physiological heterogeneity that would confound the BMI-controlled models central to the study hypothesis. Patients were included if at least one primary biochemical outcome (FT3, LDH, or ALT) was available at admission (T0). EDS-R score was used as the primary continuous exposure; two patients with missing EDS-R data (both AN-BP subtype) were retained in the overall sample (N = 164) but excluded from EDS-R score category analyses (n = 162; AN-BP in categories: n = 40) and from regression models via listwise deletion.

### 2.3. Measures

The Italian-language version of the EDS-R, whose psychometric properties and factorial validity in Italian samples have been established by Costa et al. [28], was used in this study. The EDS-R [29] is a 21-item self-report questionnaire assessing exercise dependence psychopathology across seven subscales: Withdrawal, Continuance, Tolerance, Lack of Control, Reduction in Other Activities, Time, and Continuance-Negative Affect. Each item is rated on a 6-point Likert scale (1 = never, 6 = always); scores are averaged to yield a global mean score (range 1–6). Higher scores indicate greater exercise dependence severity. The EDS-R measures the psychological dimension of exercise dependence and does not quantify objective activity volume or energy deficit. In this study, only the global mean EDS-R score was available in the clinical database; item-level data were not retained. The EDS-R was administered as part of the standardised admission psychometric battery, with clinician support (items read aloud on request; clarification of wording provided on request) without influencing response selection. No patient in this cohort presented with cognitive impairment affecting understanding of the items.

Biochemical parameters. Fasting venous blood samples were obtained within 48 h of admission (morning collection, after overnight fast), prior to initiation of any refeeding protocol, as part of routine clinical assessment. Three primary biochemical outcomes were defined a priori based on the study hypothesis. No multiplicity correction was applied to the three primary OLS regression models, which were treated as independent a priori exploratory tests on biological grounds; *p*-values are therefore nominal and should be interpreted accordingly. (1) Free triiodothyronine (FT3, pg/mL) was assessed as an indicator of thyroid axis status; (2) lactate dehydrogenase (LDH, U/L) was assessed as an indicator of cellular metabolic activity; and (3) alanine aminotransferase (ALT, U/L) was assessed as an indicator of hepatocellular activity. Secondary parameters included FT4, TSH, AST, CPK, fasting glucose, albumin, haemoglobin, white blood cell count (WBC), potassium, phosphorus, total cholesterol, vitamin D, and ferritin. Low-T3 syndrome was defined as FT3 < 2.3 pg/mL, consistent with established laboratory reference ranges and prior studies in AN [23,24].

Clinical and demographic variables. Age at admission (years), BMI (kg/m^2^), age of illness onset (years), AN subtype (AN-R vs. AN-BP), and prior hospitalisation history were extracted from clinical records. Illness duration was calculated as the difference between age at admission and age of illness onset. Missing biochemical data reflect the retrospective nature of the study: the three planned primary outcomes were available for the large majority of patients (FT3: n = 145; LDH: n = 125; ALT: n = 155; regression samples after listwise deletion: Model A n = 144, Model B n = 124, Model C n = 154). Among secondary parameters, CPK was available for 99 patients (60.4%) in the full primary cohort; 98 of these also had available EDS-R data and therefore contributed to EDS-R-based CPK analyses. CPK was not part of the standard admission panel and was ordered at the clinician’s discretion; data on this parameter are therefore reported as descriptive and interpreted with caution. Missing values were handled by listwise deletion within each model.

### 2.4. Statistical Analysis

Descriptive statistics are reported as mean ± SD or median (IQR). Normality was assessed with the Shapiro–Wilk test. Group differences across EDS-R score categories (n = 164; two patients with missing EDS-R data (both AN-BP) excluded from category analyses (AN-BP in categories: n = 40/164), leaving n = 162 with available EDS-R) were examined using the Kruskal–Wallis test with Bonferroni correction for three primary comparisons (threshold: *p* < 0.017). Spearman correlations between continuous EDS-R score and biochemical parameters were computed with Bonferroni correction for 16 simultaneous tests (threshold: *p* < 0.0031). Multivariable linear regression models were planned for three outcomes: log(FT3), LDH, and log(ALT), each adjusted for BMI, age at admission, AN subtype, and illness duration. A binary logistic regression with low-T3 syndrome (FT3 < 2.30 pg/mL, the laboratory lower reference limit for the Siemens automated immunoassay analyser) was included as a planned supplementary analysis. All unstandardised β coefficients reflect per 1-unit increase in mean EDS-R item score. Variance Inflation Factors (VIFs) were inspected to assess multicollinearity. Exploratory post hoc analyses comprised an EDS-R  ×  AN subtype interaction term and subgroup stratification by AN subtype. The primary analysis was restricted to first admissions only (n = 164); a sensitivity analysis including all 177 observations (176 AN patients; repeat admissions retained) yielded materially consistent results: log(FT3) β = −0.041 [−0.072, −0.009], *p* = 0.011, N = 155; LDH β = +15.53 [5.35, 25.71], *p* = 0.003, N = 134; log(ALT) β = +0.028 [−0.041, 0.097], *p* = 0.418, N = 166. Statistical analyses were performed using Python 3.11.9 (NumPy 1.26.4, SciPy 1.11.4, pandas 2.1.4, statsmodels 0.14.1). All tests were two-tailed; α = 0.05. Additional statistical details, including collinearity diagnostics, partial correlations controlling for BMI, AN-R vs. AN-BP subgroup comparisons, binary logistic regression models for low-T3 syndrome, and categorical BMI sensitivity analyses, are reported in Supplementary Table S1.

## 3. Results

### 3.1. Sample Characteristics

A total of 164 patients with AN (first admission per patient) (AN-R: n = 122, 74.4%; AN-BP: n = 42, 25.6%) were included. The sample was predominantly female (161 females, 98.2%; 3 males, 1.7%). Mean age at admission was 19.0 ± 5.2 years, mean BMI was 15.7 ± 1.7 kg/m^2^, and mean illness duration was 3.8 ± 4.2 years. Two patients had missing EDS-R data and were excluded from score category-based descriptive analyses; the remaining 162 patients with available EDS-R data were classified into EDS-R score categories. Based on mean EDS-R score, 70 of 162 patients with available EDS-R data (43.2%) were in the low-descriptive-score band (1–2), 44 (27.2%) in the moderate band (3–4), and 48 (29.6%) in the high-score category (5–6). BMI differed significantly across EDS-R score categories (*p* = 0.040), with lower values in the moderate- and high-score categories (16.3 ± 2.3 vs. 15.5 ± 1.5 vs. 15.4 ± 1.7 kg/m^2^), confirming the rationale for BMI adjustment in all regression models. No significant differences were observed across EDS-R score categories in age, illness duration, or AN subtype distribution. Sample characteristics are presented in Table 1.

### 3.2. Biochemical Profile Across EDS-R Score Categories

Among the 16 biochemical parameters examined, FT3 and LDH showed the largest Spearman correlations, though neither survived Bonferroni correction (corrected threshold *p* < 0.0031). FT3, LDH, and ALT were defined a priori as the primary biochemical outcomes on the basis of the study hypothesis and biological rationale. Kruskal–Wallis group comparisons for the three primary outcomes did not survive the corresponding Bonferroni-corrected threshold of *p* < 0.017 (FT3: *p* = 0.026; LDH: *p* = 0.053; ALT: *p* = 0.727) and are reported descriptively. FT3 decreased across EDS-R groups: medians were 2.4 (IQR 1.9–3.0), 2.0 (1.7–2.8), and 2.0 (1.7–2.4) pg/mL in the low EDS-R score (1–2), moderate EDS-R score (3–4), and high-score groups, respectively. The prevalence of low-T3 syndrome (FT3 < 2.3 pg/mL) increased stepwise from 45.6% in the low-score group to 60.0% in the moderate-score group and 72.9% in the high-score group. LDH medians were 297 (212–351), 325 (270–397), and 353 (299–391) U/L (Kruskal–Wallis *p* = 0.053). Spearman correlations showed a negative association between EDS-R and FT3 (ρ = −0.224, *p* = 0.007, n = 144) and a positive association between EDS-R and LDH (ρ = +0.244, *p* = 0.006, n = 124); neither survived Bonferroni correction.

In contrast, ALT showed no significant association with EDS-R score category (Kruskal–Wallis *p* = 0.727) and no Spearman correlation with EDS-R (ρ = +0.059, *p* = 0.468, n = 154). Medians across EDS-R groups were 25 (17–45), 28 (17–48), and 27 (17–58) U/L for low-EDS-R-score (1–2), moderate-EDS-R-score (3–4), and high-score patients, respectively. The pattern for all other biochemical parameters, including FT4, TSH, AST, CPK, glucose, albumin, haemoglobin, WBC, electrolytes, cholesterol, vitamin D, and ferritin, was also non-significant (all Spearman *p* > 0.20 except AST *p* = 0.218; all Kruskal–Wallis *p* > 0.12)(Table 2).

### 3.3. Multiple Regression Analyses

Table 3 presents the three planned regression models side by side, designed to render visible the contrasting associations between EDS-R-associated and BMI-associated biochemical signals. Model A (outcome: log-FT3; N = 144; R^2^adj = 0.070): EDS-R was independently and negatively associated with FT3 (β = −0.040, 95% CI [−0.072, −0.008], *p* = 0.016), while BMI was not significant (*p* = 0.434). Age at admission was also a significant predictor (β = −0.019, *p* = 0.041). The partial correlation confirmed the EDS-R and FT3 association independent of BMI (r = −0.229, *p* = 0.005). Cohen’s f^2^ for EDS-R was 0.036. VIF values for all predictors were within the acceptable range (EDS-R: 1.08; BMI: 1.10; age: 3.15; AN-BP subtype: 1.08; illness duration: 3.12), confirming the absence of problematic multicollinearity between EDS-R and BMI despite their modest correlation (Pearson r = −0.149, *p* = 0.058).

Model B (outcome: LDH; N = 124; R^2^adj = 0.036): EDS-R was the sole variable independently associated with LDH (β = +14.56, 95% CI [3.78, 25.35], *p* = 0.009). BMI, age, AN subtype, and illness duration were all non-significant (all *p* > 0.23). Each one-unit increase in mean EDS-R item score corresponded to a 14.56 U/L increase in LDH. Partial correlation: r = 0.256, *p* = 0.007. Cohen’s f^2^ = 0.072.

**Figure 1:**
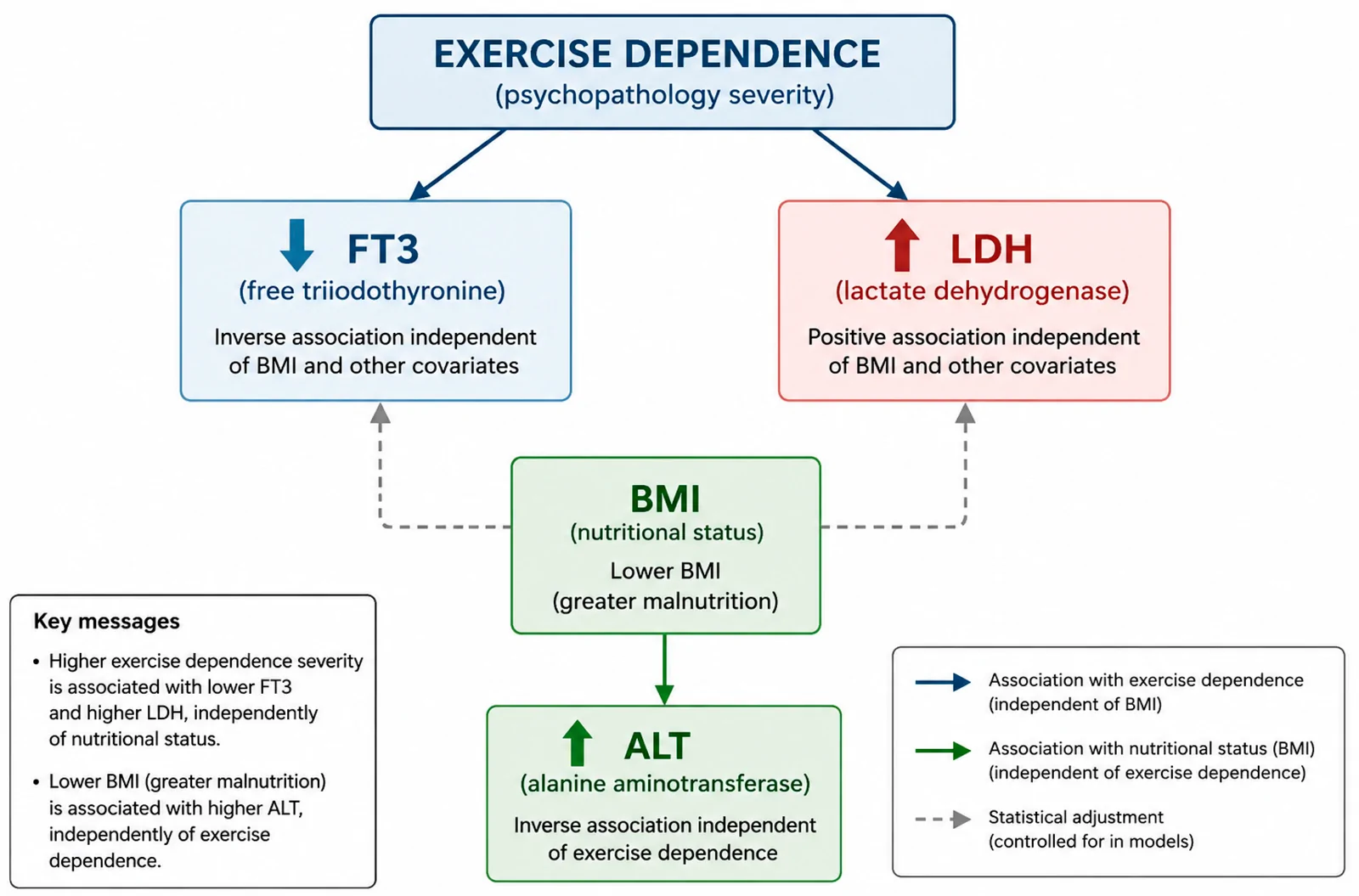
Schematic representation of the differential biochemical associations of EDS-R score and BMI in patients with anorexia nervosa at residential admission. Higher EDS-R scores showed adjusted cross-sectional associations with lower FT3 levels (model A: β = −0.040, *p* = 0.016) and higher LDH levels (model B: β = +14.56, *p* = 0.009), while BMI was not significant in either model. By contrast, higher BMI was the sole significant predictor of lower ALT (model C: β = −0.078, *p* = 0.008), with EDS-R score non-significant. All associations are adjusted cross-sectional estimates and do not establish directionality or causation. ↑ indicates values above the upper reference limit; ↓ indicates values below the lower reference limit.

Model C (outcome: log-ALT; N = 154; R^2^adj = 0.040): EDS-R was non-significant (β = 0.019, 95% CI [−0.053, 0.091], *p* = 0.604), while BMI was the sole significant predictor (β = −0.078, 95% CI [−0.136, −0.021], *p* = 0.008): higher BMI was associated with lower ALT. Partial correlation between EDS-R and ALT controlling for BMI confirmed the null association (r = 0.071, *p* = 0.360). This differential pattern is summarised in Figure 1.

### 3.4. Sensitivity and Subgroup Analyses

Sensitivity analyses replacing continuous BMI with categorical strata (<14, 14–16, >16 kg/m^2^) yielded consistent results: EDS-R remained significantly associated with FT3 (β = −0.040, *p* = 0.016) and LDH (β = +14.56, *p* = 0.009). A binary logistic regression with low-T3 syndrome (FT3 < 2.3 pg/mL) as the outcome confirmed the EDS-R association (OR = 1.290 per unit increase, 95% CI [1.043, 1.596], *p* = 0.019, n = 144), with BMI non-significant (*p* = 0.355). Exploratory subtype analyses showed an EDS-R association with FT3 in AN-R (β = −0.058, *p* = 0.001, N = 111) but not in AN-BP (β = +0.024, *p* = 0.524, N = 33). In a post hoc logistic interaction model (N = 144; 83 low-T3 cases), the EDS-R × AN-BP interaction term was significant (OR = 0.474, 95% CI [0.282, 0.797], *p* = 0.005) and directionally consistent with a stronger EDS-R association with low-T3 syndrome in AN-R; this finding is strictly exploratory. A female-only sensitivity analysis (female cohort N = 161) yielded materially unchanged estimates (log[FT3]: EDS-R β = −0.040, *p* = 0.014; LDH: EDS-R β = +14.71, *p* = 0.009; low-T3 logistic model: OR = 1.300, *p* = 0.017). These subgroup and interaction findings should be interpreted with caution given the small AN-BP sample (n = 42 total; N = 31–33 in regression models). The adjusted explained variance was modest (R^2^adj = 0.070 for FT3 and 0.036 for LDH), and none of the 16 Spearman correlations survived Bonferroni correction (threshold *p* < 0.0031). These findings are hypothesis-generating rather than confirmatory and require replication in larger, subtype-stratified cohorts.

## 4. Discussion

The central finding of this study is a pattern of differential biochemical associations in patients with AN at residential admission: higher exercise dependence psychopathology scores showed adjusted cross-sectional associations with lower FT3 and higher LDH, whereas ALT was associated with BMI rather than EDS-R score. This pattern, shown in Table 3 across the three prespecified models, suggests that EDS-R score and BMI may capture partly different correlates of the biochemical profile at admission; however, the cross-sectional design does not permit causal or mechanistic interpretation.

This differential pattern may reflect distinct physiological responses to nutritional deprivation and exercise dependence psychopathology, and suggests that EDS-R score may capture a dimension of severity at admission not adequately indexed by BMI alone. AN subtype was not an independent predictor in the adjusted models; the exploratory finding of a stronger association between EDS-R score and FT3 in AN-R than in AN-BP should be interpreted with caution given the small AN-BP subsample (n = 42 in the full cohort; N = 33 in the FT3 regression model).

A possible biological mechanism linking exercise dependence psychopathology and FT3 in severe energy deficit states involves inhibition of deiodinase enzymes responsible for converting T4 to FT3 [30,31,32]; however, this mechanism requires prospective investigation and cannot be inferred from the present cross-sectional data. The progressive stepwise increase in low-T3-syndrome prevalence across EDS-R score categories (45.6% in low EDS-R score (1–2), 60.0% in moderate EDS-R score (3–4), 72.9% in high-score patients) is consistent with a graded association: two patients with identical BMI at admission may present with different thyroid profiles depending on their exercise dependence psychopathology severity, a hypothesis consistent with our exploratory findings but not directly testable from cross-sectional data [27], a distinction that BMI-based assessment alone cannot capture.

The LDH finding is consistent with non-specific cellular or tissue stress, including starvation-induced hepatocyte injury and autophagy previously described in AN [33,34]; however, LDH is a highly non-specific enzyme elevated by multiple conditions including muscle trauma, haemolysis, and hepatic injury. The absence of a significant CPK association with EDS-R further limits interpretation of LDH as a marker of exercise-related muscle stress. CPK showed only a descriptive, non-significant pattern across EDS-R score categories (medians 52, 59, and 66 U/L for low, moderate, and high EDS-R score groups; Spearman ρ = +0.128, p = 0.209, n = 98). These observations are exploratory and require prospective replication with objective physical-activity measurement and more specific markers of tissue injury. Across the remaining biochemical parameters, most associations with EDS-R were null. This broader pattern should not be interpreted as evidence of biomarker specificity, particularly because missingness varied by parameter and none of the 16 Spearman correlations survived Bonferroni correction. From a clinical perspective, higher EDS-R scores were descriptively associated with a greater prevalence of low-T3 syndrome in this sample (72.9% in the high-score group versus 45.6% in the low-score group), but whether this observation has clinical implications requires prospective investigation. The exploratory association between EDS-R and FT3 was present in the AN-R subgroup but not in the smaller AN-BP subgroup; the significant interaction term is hypothesis-generating and requires replication in larger subtype-stratified cohorts.

Several limitations require acknowledgement. Several potentially relevant biochemical parameters were available in the dataset but not included in the primary analysis, including C-reactive protein (CRP; N = 73), transferrin (N = 67), creatinine (N = 160), haemoglobin (N = 153), and white blood cell count (N = 155). Exploratory analyses confirmed that none of these parameters showed a significant association with EDS-R (all Spearman ρ < 0.13, all *p* > 0.10), providing no evidence of a broad association between EDS-R and these additional laboratory measures; these null exploratory results should not be taken as proof of specificity or absence of residual confounding. These parameters are available for future analyses and are reported in the Supplementary Materials. The cross-sectional design precludes any inference about directionality or causality. We cannot determine whether higher EDS-R scores precede biochemical alterations, whether biochemical alterations influence exercise dependence psychopathology, or whether a third unmeasured factor, such as illness severity, anxiety, or cortisol, explains both. All associations reported here should be interpreted as exploratory and hypothesis-generating only. Effect sizes, while statistically significant and consistent, are small (Cohen’s f^2^ = 0.036 for FT3, 0.072 for LDH), and the adjusted R^2^ values of the three models range from 3.6% to 7.0%. Although the explained variance was modest, such R^2^ values are common when behavioural variables are modelled against peripheral biological markers, for which a large proportion of variance is attributable to unmeasured physiological factors such as pulsatile cortisol secretion, immediate fasting duration, hydration status, and individual metabolic heterogeneity. Accordingly, the associations between EDS-R score and biochemical parameters reported here should be interpreted as statistically reliable but quantitatively modest. These findings should not be interpreted as establishing exercise dependence as the primary determinant of any of the three biochemical parameters, but rather as identifying an independent and previously uncharacterised contribution that warrants prospective investigation with more comprehensive physiological measurement. The EDS-R was administered with clinician support, introducing potential interviewer variability. Although the EDS-R is a validated measure of exercise dependence psychopathology, it does not directly quantify objective physical activity or energy expenditure. Therefore, patients with similar EDS-R scores may engage in different volumes of physical activity, and the present findings should be interpreted as reflecting exercise dependence psychopathology rather than objectively measured exercise behaviour. The sample is predominantly female (98.2%) from a single Italian residential unit, limiting generalisability. The exclusion of BN, while methodologically justified by the physiological heterogeneity it would introduce, means this differential association model has not been tested across eating disorder diagnoses. Finally, a further limitation is the absence of leptin measurement, medication data (psychotropic agents and nutritional supplements may affect thyroid function and hepatic enzymes), and objective physical activity assessment. Leptin, secreted by adipose tissue, decreases markedly during starvation as adipose mass is lost [35], and has been proposed as a key biological mediator of compulsive exercise in AN: leptin deficiency may generate a biological drive to exercise that reinforces exercise dependence psychopathology [36,37]. Leptin also directly modulates hypothalamic–pituitary–thyroid axis function, and its deficiency may contribute to the suppression of peripheral T4-to-FT3 conversion [37,38]. We cannot therefore exclude that the observed EDS-R and FT3 association is partly mediated or confounded by leptin. Leptin was not part of the routine clinical biochemical panel at this centre and its assay was not available within the routine operational budget of the unit; this represents a mechanistic gap that future studies should address. The ALT association with BMI, while statistically significant in the log-transformed model, was modest (β = −0.078 per kg/m^2^, *p* = 0.008) and should not be overinterpreted as a robust quantitative prediction. Adjustment for BMI was chosen a priori to isolate associations independent of BMI, age, AN subtype, and illness duration; however, BMI may also lie on the causal pathway between exercise dependence and biochemical alterations.

Future prospective studies should include leptin measurement alongside EDS-R score and thyroid hormones to test whether leptin contributes to the observed EDS-R–FT3 association, while recognising that mediation cannot be inferred from the present cross-sectional data [37,38]. Objective actigraphy, detailed medication and nutritional-supplement data, LDH isoenzyme fractionation, and broader hormonal profiling (including cortisol, IGF-1, and oestradiol) would help clarify the physiological context of the observed associations. Longitudinal assessment during treatment should examine whether changes in EDS-R scores are accompanied by changes in FT3 and LDH and thereby establish temporal relationships before any clinical or therapeutic interpretation is considered. Extension to BN and ARFID populations, with appropriate physiological stratification, would test the generalisability of the findings across eating-disorder diagnoses.

## 5. Conclusions

In this exploratory cross-sectional study of 164 patients (first admissions only) with AN admitted to a residential eating disorder unit, higher EDS-R scores at admission were independently associated with lower FT3 levels (β = −0.040, *p* = 0.016) and higher LDH levels (β = +14.56, *p* = 0.009), after adjustment for age, BMI, disease duration, and AN subtype. No significant association was observed for ALT. The association with low-T3 syndrome was confirmed in the binary logistic model (OR = 1.290, 95% CI [1.043–1.596], *p* = 0.019). These findings generate hypotheses for prospective investigation into whether exercise dependence psychopathology shows associations with the biochemical profile at admission in AN after adjustment for BMI, age, AN subtype, and illness duration. Given the exploratory, retrospective, and cross-sectional design, these results cannot establish directionality or causation, and require replication in prospective studies with objective physical activity measurement.

## Figures and Tables

**Table 1:** Sample characteristics by EDS-R score category.

Variable	Low EDS-R (1–2) n = 70	Moderate EDS-R (3–4) n = 44	High EDS-R (5–6) n = 48	Total n = 164	*p*
Age at admission, years	18.3 ± 4.3	18.9 ± 4.5	20.0 ± 5.8	19.0 ± 5.2	0.151
BMI, kg/m^2^	16.3 ± 2.3	15.5 ± 1.5	15.4 ± 1.7	15.7 ± 1.7	0.040 *
Age of illness onset, years	15.1 ± 2.5	15.6 ± 2.6	15.5 ± 3.3	15.4 ± 2.9	0.671
Illness duration, years	3.2 ± 3.3	3.3 ± 2.7	4.5 ± 4.8	3.8 ± 4.2	0.564
AN-BP subtype, n (%)	14 (20.0%)	13 (29.5%)	13 (27.1%)	42 (25.6%)	0.496
EDS-R score (mean ± SD)	1.5 ± 0.5	3.5 ± 0.5	5.3 ± 0.5	3.2 ± 1.7	<0.001

Note: Data presented as mean ± SD or n (%). *p* values from Kruskal–Wallis test or chi-square test. * *p* < 0.05. Total column refers to the full primary sample (N = 164); EDS-R score category columns are based on n = 162 (two patients with missing EDS-R data excluded from category-based analyses).

**Table 2:** Biochemical parameters by EDS-R score category: medians (IQR) and Spearman correlations with EDS-R.

Variable	Low EDS-R (1–2)	Moderate EDS-R (3–4)	High EDS-R (5–6)	*p* (KW)	ρ (EDS-R)	*p* (ρ)
FT3, pg/mL †	2.4 (1.9–3.0)	2.0 (1.7–2.8)	2.0 (1.7–2.4)	0.026 *	−0.224	0.007 *
LDH, U/L †	297 (212–351)	325 (270–397)	353 (299–391)	0.053	+0.244	0.006 *
ALT, U/L ‡	25 (17–45)	28 (17–48)	27 (17–58)	0.727	+0.059	0.468
FT4, ng/dL	0.8 (0.7–1.0)	0.8 (0.7–0.9)	0.8 (0.7–0.9)	0.539	+0.047	0.574
TSH, mIU/L	1.7 (1.2–2.0)	1.5 (0.8–2.3)	1.6 (1.2–2.2)	0.799	−0.009	0.911
AST, U/L	23 (18–29)	24 (20–30)	25 (20–35)	0.478	+0.100	0.218
CPK, U/L	52 (43–69)	59 (50–70)	66 (47–85)	0.335	+0.128	0.209
Glucose, mg/dL	75 (68–82)	76 (70–81)	73 (68–79)	0.418	−0.054	0.507
Albumin, g/dL	4.5 (4.2–4.8)	4.4 (4.2–4.7)	4.5 (4.3–4.7)	0.277	−0.027	0.751
Haemoglobin, g/dL	12.8 (12.3–13.6)	12.6 (12.0–13.1)	13.1 (12.2–14.0)	0.122	+0.045	0.585
WBC (×10^3^/µL)	4.5 (3.8–5.7)	4.4 (3.7–5.3)	4.3 (3.5–5.1)	0.443	−0.072	0.377
Potassium, mEq/L	4.3 (4.1–4.5)	4.2 (4.0–4.4)	4.4 (4.2–4.5)	0.237	+0.003	0.971
Phosphorus, mg/dL	4.0 (3.7–4.3)	3.8 (3.4–4.1)	3.9 (3.5–4.3)	0.166	−0.105	0.325
Total Cholesterol, mg/dL	168 (144–201)	158 (145–181)	164 (151–191)	0.458	−0.058	0.484
Vitamin D, ng/mL	22.9 (19.1–33.0)	22.2 (15.4–26.1)	23.4 (16.5–30.1)	0.676	−0.067	0.460
Ferritin, ng/mL	52.0 (23.4–86.8)	41.7 (30.0–89.3)	50.4 (27.3–81.8)	0.987	+0.030	0.728

Note: Data presented as median (IQR). KW = Kruskal–Wallis test. ρ = Spearman’s rho (EDS-R as continuous variable). † Nominal association with EDS-R (*p* < 0.05, uncorrected); neither FT3 nor LDH survived Bonferroni correction for 16 Spearman comparisons (threshold: *p* < 0.0031). ‡ No association with EDS-R; significant association with BMI in multivariable model. * *p* < 0.05.

**Table 3:** Multiple linear regression models for log(FT3), LDH, and log(ALT): contrasting predictor patterns.

Predictor	Model A Outcome: log(FT3) N = 144, R^2^adj = 0.070	Model B Outcome: LDH (U/L) N = 124, R^2^adj = 0.036	Model C Outcome: log(ALT) N = 154, R^2^adj = 0.040
Intercept	1.061 [0.534, 1.587] *p* < 0.001 ***	194.5 [27.8, 361.2] *p* = 0.024 *	4.130 [2.996, 5.264] *p* < 0.001 ***
**EDS-R** **score †**	**β = −0.040 [−0.072, −0.008]** β * = −0.206 *p* = 0.016 *	**β = +14.56 [3.78, 25.35]** β * = 0.263 *p* = 0.009 **	β = 0.019 [−0.053, 0.091] β * = 0.046 *p* = 0.604
BMI (kg/m^2^) ‡	β = 0.011 [−0.016, 0.037] β * = 0.064 *p* = 0.434	β = 4.90 [−3.22, 13.03] β * = 0.103 *p* = 0.239	**β = −0.078 [−0.136, −0.021]** β * = −0.210 *p* = 0.008 **
Age at admission	β = −0.019 [−0.037, −0.001] β * = −0.283 *p* = 0.041 *	β = 0.10 [−6.15, 6.35] β * = 0.005 *p* = 0.975	β = 0.024 [−0.016, 0.064] β * = 0.158 *p* = 0.237
AN-BP subtype	β = 0.000 [−0.124, 0.125] β * = 0.000 *p* = 0.996	β = −10.65 [−48.87, 27.57] β * = −0.048 *p* = 0.586	β = 0.157 [−0.106, 0.421] β * = 0.091 *p* = 0.244
Illness duration	β = 0.011 [−0.012, 0.035] β * = 0.130 *p* = 0.345	β = −0.68 [−8.46, 7.11] β * = −0.028 *p* = 0.865	β = −0.043 [−0.094, 0.008] β * = −0.219 *p* = 0.102

Note: β = unstandardised regression coefficient; All models adjusted for BMI, age at admission, AN-BP subtype, and illness duration. † EDS-R is the significant predictor. ‡ BMI is the significant predictor. Log transformation applied for FT3 (Shapiro–Wilk *p* < 0.001 raw, *p* = 0.374 log) and ALT (*p* < 0.001 raw, *p* = 0.019 log). * *p* < 0.05; ** *p* < 0.01; *** *p* < 0.001.

## Data Availability

The data that support the findings of this study are available from the corresponding author (E.P.) upon reasonable request. Data are not publicly available due to patient confidentiality and institutional data governance regulations of ASL Salerno.

## Supplementary Materials

The following supporting information can be downloaded at https://www.mdpi.com/article/10.3390/brainsci16101114/s1. Table S1: Additional statistical details for transparency. Yellow fill indicates rows where EDS-R total score is a statistically significant predictor; blue fill indicates AN-R subgroup results. Bold formatting is used for section headers and column headers only. All models were adjusted for BMI, age at admission, AN subtype, and illness duration unless otherwise noted. Abbreviations: EDS-R = Exercise Dependence Scale-Revised; VIF = Variance Inflation Factor; OR = Odds Ratio; CI = Confidence Interval; IQR = Interquartile Range; NS = non-significant; AN-R = anorexia nervosa restricting subtype; AN-BP = anorexia nervosa binge-purge subtype.

## References

[1] American Psychiatric Association (2013). Diagnostic and Statistical Manual of Mental Disorders.

[2] Arcelus J., Mitchell A.J., Wales J., Nielsen S. (2011). Mortality rates in patients with anorexia nervosa and other eating disorders: A meta-analysis of 36 studies. Arch. Gen. Psychiatry.

[3] Dalle Grave R. (2011). Eating disorders: Progress and challenges. Eur. J. Intern. Med..

[4] Meyer C., Taranis L., Goodwin H., Haycraft E. (2011). Compulsive exercise and eating disorders. Eur. Eat. Disord. Rev..

[5] Pagador A.V., Olmedillas H., Quesnel D.A., Cavero-Redondo I., Fernandez-del-Valle M. (2025). Contextualising physical activity levels in patients with anorexia nervosa: A systematic review and meta-analysis. Eur. Eat. Disord. Rev..

[6] Rizk M., Kern L., Lalanne C., Hanachi M., Melchior J.C., Pichard C., Mattar L., Berthoz S., Godart N., EVHAN Group (2019). High-intensity exercise is associated with a better nutritional status in anorexia nervosa. Eur. Eat. Disord. Rev..

[7] Cuesta-Zamora C., González-Martí I., García-López L.M., Ros L., Plateau C.R., Ricarte J.J. (2021). Emotion Dysregulation as a Mediator of the Relationship between Anxiety, Compulsive Exercise and Eating Disorder Symptoms in Adolescents. Children.

[8] Hallward L., Di Marino A., Duncan L.R. (2022). A systematic review of treatment approaches for compulsive exercise in eating disorders. Eat. Disord..

[9] Bratland-Sanda S., Sundgot-Borgen J., Rø Ø., Rosenvinge J.H., Hoffart A., Martinsen E.W. (2010). Physical activity and exercise dependence during inpatient treatment of longstanding eating disorders: An exploratory study of excessive and non-excessive exercisers. Int. J. Eat. Disord..

[10] Danielsen M., Rø Ø., Romild U., Bjørnelv S. (2016). Impact of female adult eating disorder inpatients’ attitudes to compulsive exercise on outcome at discharge and follow-up. J. Eat. Disord..

[11] Toutain M., Gauthier A., Leconte P. (2022). Exercise therapy in the treatment of anorexia nervosa: Its effects depending on the type of physical exercise—A systematic review. Front. Psychiatry.

[12] Duriez P., Guy-Rubin A., Kaya Lefèvre H., Gorwood P. (2022). Morphing analysis of facial emotion recognition in anorexia nervosa: Association with physical activity. Eat. Weight Disord..

[13] Rautou P.E., Cazals-Hatem D., Moreau R., Francoz C., Feldmann G., Lebrec D., Ogier–Denis É., Bedossa P., Valla D., Durand F. (2008). Acute liver cell damage in patients with anorexia nervosa: A possible role of starvation-induced hepatocyte autophagy. Gastroenterology.

[14] Biolato M., Terranova R., Policola C., Pontecorvi A., Gasbarrini A., Grieco A. (2024). Starvation hepatitis: Clinical and histological features and its management. Gastroenterol. Rep..

[15] Restellini S., Spahr L., Rubbia Brandt L. (2013). Severe Starvation-Induced Hepatocyte Autophagy as a Cause of Acute Liver Injury in Anorexia Nervosa: A Case Report. Case Rep. Hepatol..

[16] Cuntz U., Voderholzer U. (2022). Liver Damage Is Related to the Degree of Being Underweight in Anorexia Nervosa and Improves Rapidly with Weight Gain. Nutrients.

[17] Hanachi M., Melchior J.C., Crenn P. (2013). Hypertransaminasemia in severely malnourished adult anorexia nervosa patients: Risk factors and evolution under enteral nutrition. Clin. Nutr..

[18] Torstveit M.K., Fahrenholtz I.L., Lichtenstein M.B., Stenqvist T.B., Melin A.K. (2019). Exercise dependence, eating disorder symptoms and biomarkers of Relative Energy Deficiency in Sport (RED-S) among male endurance athletes. BMJ Open Sport Exerc. Med..

[19] Areta J.L., Taylor H.L., Koehler K. (2021). Low energy availability: History, definition and evidence of its endocrine, metabolic and physiological effects in prospective studies in females and males. Eur. J. Appl. Physiol..

[20] Støving R.K., Andersen M., Flyvbjerg A., Frystyk J., Hangaard J., Hansen-Nord M., Hagen C. (1999). Low serum levels of free and total insulin-like growth factor I (IGF-I) in patients with anorexia nervosa. J. Clin. Endocrinol. Metab..

[21] Loucks A.B., Kiens B., Wright H.H. (2011). Energy availability in athletes. J. Sports Sci..

[22] Moshang T., Parks J.S., Baker L., Vaidya V., Utiger R.D., Bongiovanni A.M., Snyder P.J. (1975). Low serum triiodothyronine in patients with anorexia nervosa. J. Clin. Endocrinol. Metab..

[23] Onur S., Haas V., Bosy-Westphal A., Hauer M., Paul T., Nutzinger D., Klein H., Müller M.J. (2005). L-tri-iodothyronine is a major determinant of resting energy expenditure in underweight patients with anorexia nervosa and during weight gain. Eur. J. Endocrinol..

[24] Wronski M.L., Tam F.I., Seidel M., Mirtschink P., Poitz D.M., Bahnsen K., Steinhäuser J.L., Bauer M., Roessner V., Ehrlich S. (2022). Associations between pituitary-thyroid hormones and depressive symptoms in individuals with anorexia nervosa before and after weight-recovery. Psychoneuroendocrinology.

[25] Fazeli P.K., Klibanski A. (2014). Anorexia nervosa and bone metabolism. Bone.

[26] Lawson E.A., Klibanski A. (2008). Endocrine abnormalities in anorexia nervosa. Nat. Clin. Pract. Endocrinol. Metab..

[27] Usdan L.S., Khaodhiar L., Apovian C.M. (2008). The endocrinopathies of anorexia nervosa. Endocr. Pract..

[28] Mountjoy M., Sundgot-Borgen J.K., Burke L.M., Ackerman K.E., Blauwet C., Constantini N., Lebrun C., Lundy B., Melin A.K., Meyer N.L. (2018). IOC consensus statement on relative energy deficiency in sport (RED-S): 2018 update. Br. J. Sports Med..

[29] Hausenblas H.A., Downs D.S. (2002). How much is too much? The development and validation of the Exercise Dependence Scale. Psychol. Health.

[30] Kheloufi M., Boulanger C.M., Durand F., Rautou P.E. (2014). Liver autophagy in anorexia nervosa and acute liver injury. BioMed Res. Int..

[31] Rosen E., Bakshi N., Watters A., Rosen H.R., Mehler P.S. (2017). Hepatic complications of anorexia nervosa. Dig. Dis. Sci..

[32] Loucks A.B., Thuma J.R. (2003). Luteinizing hormone pulsatility is disrupted at a threshold of energy availability in regularly menstruating women. J. Clin. Endocrinol. Metab..

[33] Hackney A.C., Lane A.R. (2015). Exercise and the regulation of endocrine hormones. Prog. Mol. Biol. Transl. Sci..

[34] Hackney A.C., Viru A. (1999). Twenty-four-hour cortisol response to multiple daily exercise sessions of moderate and high intensity. Clin. Physiol..

[35] Manuelli M., Blundell J.E., Biino G., Cena H. (2019). Body composition and resting energy expenditure in women with anorexia nervosa: Is hyperactivity a protecting factor?. Clin. Nutr. ESPEN.

[36] Himmerich H., Keeler J.L., King J.A., Ehrlich S., Kaufmann L.-K., Bulik C.M., Cohen-Woods S., Wade T., Steiger H., Booij L. (2026). World Federation of Societies of Biological Psychiatry (WFSBP) consensus statement on candidate biomarkers for anorexia nervosa. World J. Biol. Psychiatry.

[37] Hebebrand J., Exner C., Hebebrand K., Holtkamp C., Casper R.C., Remschmidt H., Herpertz-Dahlmann B., Klingenspor M. (2003). Hyperactivity in patients with anorexia nervosa and in semistarved rats: Evidence for a pivotal role of hypoleptinemia. Physiol. Behav..

[38] Monteleone P., Fabrazzo M., Tortorella A., Fuschino A., Maj M. (2002). Opposite modifications in circulating leptin and soluble leptin receptor across the eating disorder spectrum. Mol. Psychiatry.

