# Supplementary material for: Exercise Dependence Psychopathology Is Associated with Differential Biochemical Correlates at Admission in Anorexia Nervosa: An Exploratory Cross-Sectional Study
Source: Brain Sci. 2026 Oct 15;16(10):1114. doi: 10.3390/brainsci16101114 (PMC13702202; doi:10.3390/brainsci16101114)
Supplement: Supplementary file 1 [file brainsci-16-01114-s001.zip › brainsci-4542276-supplementary.pdf]

Supplementary Table S1. Extended Statistical Analyses

S1.1 Collinearity Diagnostics for EDS-R and BMI

Predictor	VIF	Tolerance	Condition Index	Interpretation
EDS-R total score	1.04	0.962	—	Negligible collinearity
BMI (kg/m²)	1.04	0.962	—	Negligible collinearity
Model eigenvalue 1	—	—	1.000	Reference
Model eigenvalue 2	—	—	1.042	Well below threshold (< 15)

VIF = Variance Inflation Factor. VIF > 10 and/or Condition Index > 30 indicate problematic collinearity. All values are within acceptable limits.

S1.2 Partial Correlations (EDS-R vs. Hormonal/Metabolic Variables), Controlling for BMI

Variable	Partial r	95% CI	p-value	Interpretation
FT3 (pg/mL)	−0.317	[−0.484, −0.132]	0.001	Significant inverse association
FT4 (pg/mL)	−0.098	[−0.294, 0.104]	0.339	Non-significant
TSH (µIU/mL)	−0.011	[−0.211, 0.190]	0.914	Non-significant
FT3/FT4 ratio	−0.273	[−0.447, −0.083]	0.005	Significant inverse association
Cortisol (µg/dL)	0.144	[−0.057, 0.336]	0.158	Non-significant
IGF-1 (ng/mL)	−0.189	[−0.378, 0.008]	0.060	Trend (marginal)
Total T3/FT4 ratio	−0.261	[−0.437, −0.070]	0.008	Significant inverse association

*Partial correlations computed with BMI as covariate (N = 97 complete cases). Yellow rows: EDS-R shows a statistically significant association (p < 0.05). 95% CI derived via Fisher z-transformation. ~ p < 0.10; * p < 0.05; ** p < 0.01.*

S1.3 AN-R vs. AN-BP Subgroup Sensitivity Analysis

Variable	AN-R (n = 64–66) Median [IQR]	AN-BP (n = 31–33) Median [IQR]	p-value	Test
EDS-R total score	55.2 [46.0–64.5]	58.7 [49.0–68.3]	0.214	Mann-Whitney U
FT3 (pg/mL)	2.8 [2.3–3.2]	2.7 [2.2–3.1]	0.481	Mann-Whitney U
FT3/FT4 ratio	0.44 [0.37–0.52]	0.41 [0.35–0.49]	0.308	Mann-Whitney U
Low-T3 syndrome prevalence	71.2%	75.8%	0.621	χ^2
BMI (kg/m²)	15.8 [14.9–17.1]	15.4 [14.4–16.8]	0.341	Mann-Whitney U
Cortisol (µg/dL)	16.4 [12.1–22.3]	17.9 [13.5–24.6]	0.412	Mann-Whitney U
IGF-1 (ng/mL)	68.3 [51.2–89.4]	64.1 [48.7–84.2]	0.527	Mann-Whitney U

Blue rows: AN-R subgroup analyses. AN-BP subgroup (n = 31–33) is underpowered; interpret all comparisons as exploratory only. p-values from Mann-Whitney U test or χ^2 as indicated. No significant subgroup differences were observed for any variable.

S1.4 Binary Logistic Regression: Predictors of Low-T3 Syndrome (FT3 < 2.5 pg/mL)

Predictor	B	SE	Wald χ^2	p-value	OR	95% CI (OR)
EDS-R total score	0.061	0.019	10.33	0.001	1.063	[1.026, 1.101]
BMI (kg/m²)	−0.287	0.087	10.88	0.001	0.751	[0.633, 0.891]
Cortisol (µg/dL)	0.023	0.017	1.83	0.176	1.023	[0.990, 1.058]
IGF-1 (ng/mL)	−0.004	0.005	0.64	0.424	0.996	[0.986, 1.006]
Constant	4.218	1.532	7.58	0.006	—	—

Model fit: Nagelkerke R^2 = 0.312; Hosmer-Lemeshow $\chi^2(8)$ = 6.41, p = 0.602 (good fit). Classification accuracy = 74.2%. Yellow row: EDS-R is a statistically significant predictor. OR = Odds Ratio; CI = Confidence Interval.

S1.4b Post-hoc Model with EDS-R × BMI Interaction Term

Predictor	B	SE	Wald χ^2	p-value	OR	95% CI (OR)
EDS-R total score	0.059	0.021	7.88	0.005	1.061	[1.018, 1.106]
BMI (kg/m²)	−0.291	0.093	9.79	0.002	0.747	[0.622, 0.898]
EDS-R × BMI interaction	−0.001	0.004	0.06	0.806	0.999	[0.991, 1.007]
Cortisol (µg/dL)	0.022	0.017	1.67	0.196	1.022	[0.989, 1.057]
Constant	4.361	1.698	6.60	0.010	—	—

Interaction term EDS-R × BMI is non-significant (p = 0.806), confirming that the association between EDS-R and low-T3 syndrome is not moderated by BMI. Model fit: Nagelkerke R^2 = 0.313.

S1.5 Sensitivity Analysis: Logistic Regression with Categorical BMI Strata

Predictor	B	SE	Wald χ^2	p-value	OR	95% CI (OR)
EDS-R total score	0.058	0.020	8.41	0.004	1.060	[1.019, 1.102]
BMI stratum: 15.0–16.4 kg/m² (ref: < 15.0)	0.612	0.489	1.56	0.211	1.844	[0.707, 4.811]
BMI stratum: ≥ 16.5 kg/m²	−0.834	0.521	2.56	0.110	0.434	[0.156, 1.207]
Cortisol (µg/dL)	0.025	0.018	1.94	0.163	1.025	[0.990, 1.062]
IGF-1 (ng/mL)	−0.004	0.005	0.58	0.447	0.996	[0.986, 1.006]
Constant	2.104	1.218	2.98	0.084	—	—

BMI strata defined by tertiles: < 15.0, 15.0–16.4, ≥ 16.5 kg/m². Reference category: BMI < 15.0 kg/m². Results confirm that the association between EDS-R and low-T3 syndrome remains significant (p = 0.004) when BMI is entered as categorical rather than continuous. Model fit: Nagelkerke R^2 = 0.308. Hosmer-Lemeshow $\chi^2(8)$ = 7.12, p = 0.523.

*Legend: ~ p < 0.10; * p < 0.05; ** p < 0.01. Yellow fill = EDS-R statistically significant predictor. Blue fill = AN-R subgroup result. AN-BP subgroup (n = 31–33): underpowered — interpret as exploratory only. VIF = Variance Inflation Factor; OR = Odds Ratio; CI = Confidence Interval; IQR = Interquartile Range.*
